# Interprofessional education to implement patient falls education in hospitals: Lessons learned

**DOI:** 10.1002/nop2.1276

**Published:** 2022-06-23

**Authors:** Louise Shaw, Debra Kiegaldie, Hazel Heng, Meg. E. Morris

**Affiliations:** ^1^ Faculty of Health Science, Youth and Community Studies Holmesglen Institute Moorabbin Victoria Australia; ^2^ School of Allied Health, Human Services and Sport La Trobe University Bundoora Victoria Australia; ^3^ Eastern Health Clinical School Monash University Melbourne Victoria Australia; ^4^ Healthscope Holmesglen Private Hospital Moorabbin Victoria Australia; ^5^ Academic and Research Collaborative in Health La Trobe University Bundoora Victoria Australia; ^6^ Northern Health Epping Victoria Australia; ^7^ Victorian Rehabilitation Centre Glen Waverly Victoria Australia; ^8^ College of Healthcare Sciences James Cook University Douglas Queensland Australia

**Keywords:** accidental falls, hospital, implementation, interprofessional education, nursing, physiotherapy, simulation

## Abstract

**Aim:**

The aim of this study was to design, deliver and evaluate an interprofessional education programme for healthcare professionals on how to implement a modified version of the safe recovery programme to prevent falls in hospitalized patients.

**Design:**

Mixed methods design incorporating pre‐ and post education surveys and individual semi‐structured interviews.

**Methods:**

Thirty‐four health professional participants attended a 1‐h face‐to‐face or Zoom® interprofessional education session to learn how to deliver an evidence‐based patient falls prevention education strategy, the modified Safe Recovery Programme.

**Results:**

A 1‐hour education session was insufficient to build full confidence to deliver the Safe Recovery Programme. There was no statistically significant change in participant views on interprofessional collaboration. Participants recommended prior consultation and preparation before delivery of IPE, with additional opportunities for discussion and feedback during implementation with patients. The findings highlight the importance of interprofessional education for evidence‐based interventions in hospitals. Health professionals value education that is timely, interactive, realistic and engaging.

## 1 INTRODUCTION

Despite a wide range of interventions for preventing falls in hospitals and care facilities, falls in healthcare organizations remain a serious problem worldwide (Avanecean et al., 2017; Cameron et al., 2018; Morris et al., 2021). Patient education is an integral part of falls prevention and has been found to have a positive effect on the risk of hospital falls (Francis‐Coad et al., 2021; Heng, Jazayeri, et al., 2020; Hill et al., 2019; Hill, McPhail, Waldron, Etherton‐Beer, Flicker, et al., 2015; Hill, McPhail, Waldron, Etherton‐Beer, Ingram, et al., 2015). Falls prevention education for patients aims to increase their understanding of falls and falls risk, as well as empowering them with falls prevention strategies (Haines et al., 2015; Naseri et al., 2021).

## 2 BACKGROUND

The Safe Recovery Programme (SRP) is an individualized patient education programme based on the principles of health behaviour change and has been found to be effective in reducing falls rates in hospitals (Haines et al., 2011; Hill, McPhail, Waldron, Etherton‐Beer, Flicker, et al., 2015; Hill, McPhail, Waldron, Etherton‐Beer, Ingram, et al., 2015). The SRP has four stages aimed at empowering patients to improve their safety in hospital: (i) assessing risks; (ii) goal setting; (iii) reviewing goals; and (iv) follow‐up (Haines et al., 2011; Hill, McPhail, Waldron, Etherton‐Beer, Flicker, et al., 2015; Hill, McPhail, Waldron, Etherton‐Beer, Ingram, et al., 2015).

Patients have been found to prefer individualized education as well as consistent and standardized information from all clinical staff (Heng, Slade, et al., 2020; Naseri et al., 2021). Allied health professionals and nurses play a key role in providing patient education (Hill et al., 2016; Shaw et al., 2020; Shaw, Kiegaldie, & Morris, 2021; Shaw, Kiegaldie, Morris, & Jones, 2021), making it essential to educate them on how to do this effectively. Education programmes for healthcare professionals that embed evidence‐based practice as well as evidence‐based teaching and learning methods enhance quality patient care (Lehane et al., 2019). High‐quality, interactive education programmes for healthcare professionals are known to influence implementation of falls prevention strategies in a hospital setting (Shaw et al., 2020; Shaw, Kiegaldie, & Morris, 2021; Shaw, Kiegaldie, Morris, & Jones, 2021). Effective implementation of falls prevention interventions necessitates the creation of a positive learning environment that increases receptivity to a new intervention (Shaw et al., 2020; Shaw, Kiegaldie, & Morris, 2021; Shaw, Kiegaldie, Morris, & Jones, 2021).

**FIGURE 1:**
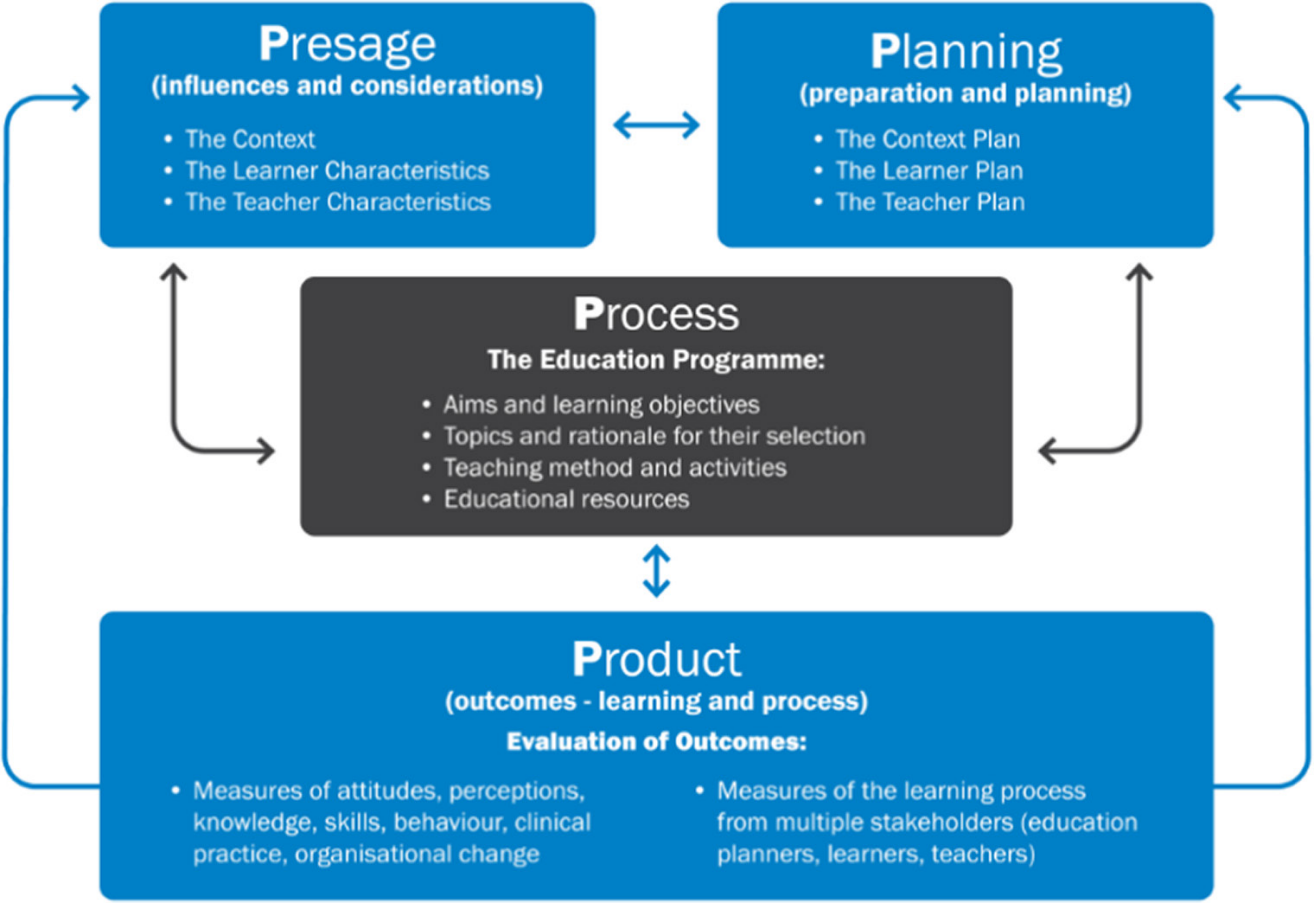
4P model of educational design (Kiegaldie, 2015)

Interprofessional education (IPE) offers opportunities for participants from different professional groups to learn with, from and about each other (Barr, 2002; Kiegaldie, 2021) to improve collaborative practice and patient‐centred care. To optimize falls prevention education to patients and ensure the content and delivery of patient education is consistent across professions, education for healthcare professionals should reflect the importance of an interprofessional approach (Heng et al., 2021; McKenzie et al., 2017; Reeves et al., 2017; Wheeler et al., 2018). Biggs' 3P model for educational design has been expanded to a 4P approach (Planning, Presage, Process and Product) (Kiegaldie, 2017). See Figure 1. The 4P model offers a comprehensive systems‐based theoretical model of learning and allows for a greater understanding of the different factors that impact educational delivery and implementation (Baker et al., 2010; Kiegaldie, 2021).

The aim of this study was to design, deliver and evaluate an IPE programme for healthcare professionals on how to implement a modified version of the SRP to prevent falls in hospitalized patients. The intervention from this study supported a trial that involved healthcare professionals delivering the modified SRP to patients in an acute hospital setting. We sought to investigate whether an IPE programme develops healthcare professionals': (i) motivation and confidence in facilitating an interactive patient education intervention on falls prevention, (ii) appreciation of the value and role of other health professionals in falls prevention, (iii) knowledge and capability for interprofessional collaboration.

## 3 METHODS

### 3.1 Study design

The study employed a mixed methods pre‐ and post questionnaire design followed by semi‐structured telephone interviews, to triangulate the data from different approaches. Sequential exploratory design was used with the interview data building on the survey results (Creswell et al., 2003).

### 3.2 Sample and study settings

The study took place on the medical wards of an Australian private acute hospital. All allied health professionals and nurses working on the intervention ward were eligible and invited to participate via email. On the day of the IPE intervention, consenting participants completed a PICF, pre‐test and post‐test surveys.

### 3.3 Intervention

Training was delivered either face to face or via a video conferencing system, Zoom®. Participants received a 1‐h IPE programme, which was the most time available for busy clinicians to attend. The programme educated participants on the latest evidence on patient education for falls prevention, how to implement the modified SRP and how to achieve effective interprofessional collaborative practice. A mixture of interactive teaching methods was used including small group discussion on participants' current falls prevention education to patients, a small group critical thinking activity on the barriers and facilitators to delivering falls prevention education to patients, and content delivery on the latest evidence on patient education and its role in falls prevention in hospitals. Three pre‐recorded vignettes using simulated participants demonstrated delivery of the modified SRP. Laminated scripts of the modified SRP were provided for participants to use during the education intervention and instructions for implementation (see Supplementary Material). Multiple copies were also available on the hospital ward throughout implementation of the intervention. The PowerPoint presentation of the education was made available for those staff unable to attend training and to supplement learning for staff who attended.

### 3.4 Ethics approval

Research Ethics Committee approval was obtained from La Trobe University’s Health and Engineering College Human Ethics Sub‐Committee (HEC21023).

### 3.5 Data collection and instruments

A pre‐test, post‐test method of data collection was used. A commercial online survey software program, Qualtrics (Qualtrics Provo, Utah, USA), was used for online data collection. Prior to receiving the IPE programme, consenting participants responded to a survey to measure their attitudes and perceptions towards evidence‐based falls prevention education and interprofessional collaborative practice. The survey on evidence‐based falls education (Table 4) includes 8 items on a five‐point Likert Scale (1 = strongly disagree, 5 = strongly agree). The Interprofessional Collaborative Competency Attainment Survey (ICCAS) (MacDonald et al., 2010) (Table 2) includes 20 items on a five‐point Likert scale (1 = Poor, 5 = Excellent). The post education survey included repeated items and an additional set of questions exploring their views and perceptions of the education programme (7 items on a five‐point Likert scale (strongly disagree = 1, strongly agree = 5) (Table 3). Following the IPE programme and implementation of the SRP patient education trial (4 weeks), individual telephone interviews were conducted with a sample of consenting participants and a representative from hospital management (Table 1), where they elaborated on the IPE and interprofessional collaborative practice strategies used during the SRP trial.

### 3.6 Data analysis

#### 3.6.1 Quantitative data

The demographic make‐up of the participants was characterized by simple frequency statistics. Participant responses to Likert scale survey questions were summarized using basic descriptive statistics (n, mean score, standard deviation and 95% confidence interval). Independent Samples t‐tests were used to compare the means. A *p‐*value <.05 was considered to be statistically significant. SPSS (IBM SPSS Statistics for Windows, version 21.0) was used to analyse the data.

#### 3.6.2 Qualitative data

Interviews were transcribed verbatim, transferred into Excel and analysed thematically (Braun & Clarke, 2006). One researcher developed initial descriptive themes and subthemes using the relevant aspects of the 4P model for IPE design (Kiegaldie, 2021). A second researcher reviewed the initial themes and the two researchers discussed and finalized the framework for analysis. Two components of Presage (Context and Learners) and four components of Process‐Education Design (content, teaching approaches, methods of delivery and educational resources) were deemed relevant for evaluating the results.

## 4 RESULTS

Table 1 presents participant demographics. Tables 2, 3, 4 present the results with pertinent quantitative data integrated into the Presage and Process components of the 4P model. Table 5 outlines the qualitative data according to the themes of Presage and Process and describes participants' views of the education intervention according to enhancing and limiting factors.

**TABLE 1:** Participant characteristics and demographics

Participant characteristics	Number of participants
Completed pre‐education surveys	34
Registered Nurse	21
Enrolled Nurse	9
Physiotherapist	2
Occupational Therapist	1
Unknown	1
Gender	
Female	26
Male	6
Unknown	2
Completed posteducation survey	25
Interview participants	
Registered Nurse	1
Enrolled Nurse	3
Physiotherapist	1
Assistant Nurse Unit Manager	1
Executive staff member	1

**TABLE 2:** Views on interprofessional collaboration pre‐ and posteducation (1 = Poor to 5 = Excellent)

Question	Pre‐/Post‐	N	Mean	Std. Dev.	95% CI	Independent samples t‐test		
						t	df	Sig.
1 Promote effective communication amongst members of an interprofessional (IP) team	PRE	34	3.59	0.70	0.24			
	POST	25	3.88	0.73	0.29	−1.548	50.846	0.128
2 Actively listen to IP team members' ideas and concerns	PRE	34	4.00	0.85	0.29	0.193	55.492	0.848
	POST	25	3.96	0.73	0.29			
3 Express my ideas and concerns without being judgmental	PRE	34	3.65	0.85	0.29	−0.875	52.751	0.386
	POST	24	3.83	0.76	0.31			
4 Provide constructive feedback to IP team members	PRE	33	3.45	0.94	0.33	−1.311	54.580	0.195
	POST	25	3.76	0.83	0.33			
5 Express my ideas and concerns in a clear, concise manner	PRE	34	3.56	0.66	0.23	−1.578	50.623	0.121
	POST	25	3.84	0.69	0.28			
6 Seek out IP team members to address issues	PRE	33	3.70	0.77	0.27	−1.149	54.014	0.256
	POST	25	3.92	0.70	0.28			
7 Work effectively with IP team members to enhance care	PRE	34	3.79	0.77	0.26	−0.806	51.118	0.424
	POST	25	3.96	0.79	0.32			
8 Learn with, from and about IP team members to enhance care	PRE	33	3.91	0.77	0.27	−0.490	55.294	0.626
	POST	25	4.00	0.65	0.26			
9 Identify and describe my abilities and contributions to the IP team	PRE	34	3.65	0.73	0.25	−0.988	51.375	0.328
	POST	25	3.84	0.75	0.30			
10 Be accountable for my contributions to the IP team	PRE	34	3.74	1.05	0.36	−0.807	56.546	0.423
	POST	25	3.92	0.70	0.28			
11 Understand the abilities and contributions of IP team members	PRE	33	3.82	0.73	0.25	−0.494	48.548	0.623
	POST	25	3.92	0.81	0.32			
12 Recognize how others' skills and knowledge complement and overlap with my own	PRE	34	3.76	0.99	0.34	−1.067	56.978	0.290
	POST	25	4.00	0.71	0.28			
13 Use an IP team approach with the patient to assess the health situation	PRE	34	3.71	0.80	0.27	−1.216	52.214	0.230
	POST	25	3.96	0.79	0.32			
14 Use an IP team approach with the patient to provide whole person care	PRE	34	3.71	0.80	0.27	−1.216	52.214	0.230
	POST	25	3.96	0.79	0.32			
15 Include the patient/family in decision‐making	PRE	34	3.91	0.93	0.32	−1.176	56.996	0.244
	POST	25	4.16	0.69	0.28			
16 Actively listen to the perspectives of IP team members	PRE	34	3.88	0.77	0.26	−0.788	50.429	0.434
	POST	24	4.04	0.75	0.31			
17 Take into account the ideas of IP team members	PRE	33	3.94	0.70	0.25	−0.526	50.640	0.601
	POST	25	4.04	0.73	0.29			
18 Address team conflict in a respectful manner	PRE	34	3.74	0.67	0.23	−0.930	45.478	0.357
	POST	25	3.92	0.81	0.32			
19 Develop an effective care plan with IP team members	PRE	33	3.76	0.66	0.23	−0.853	47.323	0.398
	POST	24	3.92	0.72	0.29			
20 Negotiate responsibilities in overlapping scopes of practice	PRE	34	3.76	0.74	0.25	−0.573	50.266	0.569
	POST	25	3.88	0.78	0.31			

**TABLE 3:** Descriptive statistics for the responses to questions on clinician education (1 = strongly disagree to 5 = strongly agree)

Question	N	Mean	Std. dev.	95% CI
1 The education was well designed	24	3.96	.751	0.31
2 It was pitched at the appropriate level	24	4.04	.624	0.25
3 It was delivered to a high standard	22	3.82	.907	0.39
4 It was taught in an interactive and engaging manner	24	3.96	.690	0.28
5 The learning resources were useful	24	3.71	.690	0.28
6 I feel prepared to educate patients about falls prevention	23	4.04	.562	0.23
7 I am satisfied with the skills gained from this education session	24	4.04	.690	0.28

**TABLE 4:** Views on evidence‐based education for falls prevention (1 = strongly disagree to 5 = strongly agree)

Question	Pre‐/Post‐	N	Mean	Std. Dev.	95% CI	Independent samples t‐test		
						t	df	Sig.
Undertaking training to teach patients about falls prevention will be useful to me	PRE	34	4.41	0.66	0.23	0.843	56.186	0.403
	POST	25	4.28	0.54	0.22			
I am aware of current best practice relating to patient education for the prevention of falls in hospitals	PRE	34	3.94	0.60	0.21	−1.720	55.709	0.091
	POST	25	4.16	0.37	0.15			
It is important to me that the education I deliver to patients on falls prevention is based on the best available evidence	PRE	34	4.68	0.53	0.18	1.141	53.213	0.259
	POST	25	4.52	0.51	0.20			
I feel confident promoting evidence‐based strategies for patient education on falls prevention education to colleagues	PRE	34	3.82	0.80	0.27	−1.630	56.951	0.109
	POST	25	4.12	0.60	0.24			
I believe I am able to learn new strategies for educating patients on falls prevention whilst in hospital	PRE	34	4.32	0.77	0.26	1.326	56.023	0.190
	POST	25	4.08	0.64	0.26			
I feel well prepared for educating patients on falls prevention in my clinical practice area	PRE	34	3.76	0.70	0.24	−2.789	56.974	0.007**
	POST	25	4.20	0.50	0.20			
I believe that I can overcome barriers to implementing patient education on falls prevention in my clinical area	PRE	34	3.85	0.82	0.28	0.585	49.362	0.561
	POST	25	3.72	0.89	0.36			
My colleagues believe delivering evidence‐based falls prevention education to patients is an important part of our role	PRE	34	4.03	0.80	0.27	−0.867	54.929	0.390
	POST	25	4.20	0.71	0.28			

^**^ Significance level at the *p* < 0.05 level.

**TABLE 5:** Qualitative themes based on the 3P approach to IPL

Themes	Perceptions of learning experience	
	Enhancing factors (what was done well)	Limiting factors (suggestions for improvement)
**Presage components: The context**		
1 Relationship with stakeholders: Consultation and pre‐course preparation		Consultation and explanation required prior to initial education session to facilitate on‐boarding. Regular “lead in” updates during ward handover. “…I think the best way to go about introducing it, probably would be… an initial onboarding orientation to the facility that’s very clear, ‘This is how we do it here, this is the model that we take as our approach’, and then providing ongoing refreshment in‐services.” (P3)
2 Management support: Time available for education		Longer initial session more beneficial “I think actually [immersing in] the educational task, even a longer interactive session, it’s worth it. Like, you can roster staff on their off days and maybe get them paid two hours just to get it done, if it’s going to ‐ if we know that the evidence is there.” (P2)
		Initial session needs to be followed up with at least one or more refresher sessions. “…doing one – the in‐service and then maybe two or three weeks later doing a refresher in‐service, might be helpful just to say, ‘OK, we’ve been doing this for two weeks now, just let’s quickly recap how are you finding it, do you need support?’ I think that would probably be a good way to do it.” (P3)
		Deliver as on‐ward in‐service training outside of working shift. “I think it’s probably cheaper to have us stay for half an hour, than to pay for X number of falls per year. So, having the support of staff staying a little bit…I would stay an extra half an hour to enable other staff on my ward to attend an in‐service.” (P3)
3 Learning environment: Online versus face to face	The ability to complete the training online. “Look, doing shift work … doing the Zoom ones actually worked a lot better, I think. It is something that corona has given to the world is the ability to actually do this, whereas people were resistant to that before.” (P7)	Face‐to‐face delivery to allow full interaction of clinicians. “I think it would have been more valuable as an in‐person thing, but that’s just because the way that I learn, I like face‐to‐face learning… I was just sitting in my lounge room, not as engaged as I probably could have been.” (P3)
	Face‐to‐face training “I mean, all in all, the education was thorough enough that I was able to get what I needed to out of it, to then implement it to the best of my ability on the ward.” (P3)	Accessibility of training online followed by interactive face‐to‐face discussion “I would do regular discussion groups, but short. I would have the bulk of the information done online, and then I would have, maybe after they’ve completed that, a 10, 15 minute discussion, very quick, and then a week or two weeks, another little discussion, how you going, what can we improve on?” (P5)
4 The learners: Interprofessional collaboration	Existing intra and interprofessional communication further reinforced “I think behaviour that helped was everyone being involved. I mean, we were all educated about it, around about the same time, so we all sort of – I guess we were on the same page, which I think was more or less a protective factor in terms of implementing the work.” (P3)	No real change in intra and interprofessional collaboration posttraining “‘I didn’t have a lot of discussions about this programme, but allied health is always discussing with us nurses about the mobility and falls risk and so forth of the patient. I didn’t really have the discussions with them about it. I didn’t notice it [change in communication], so I think it’s maybe stayed the same.” (P5)
**Process: The Education Programme**		
1 Content: Evidence‐based practice	Increased confidence and motivation to implement “…for me, being able to be involved in something like this, as well as I guess, putting into practice having evidence‐based care, as a clinician that’s personally important. I think the development of evidence‐based care is essential to nursing. I mean, it’s the only way we get anywhere really. So, it was good, I suppose, knowing that that’s what it was.” (P3)	Already confident the care given was evidence‐based therefore no impact “I didn’t have any concerns about what I was doing in the past, because I was using evidence to the best of my knowledge, so I wasn’t using this any more than the things I’d done in the past before.” (P1)
2 Teaching approaches and activities	Simulation videos demonstrating the intervention “…yeah, it was like a real‐life scenario, hearing it actually out loud and sort of being role played, yeah, it showed how the conversation can flow and it showed how it can work.”	Training scenarios need to be more realistic, for example hearing difficulties, language barriers “…with the role plays …a little bit less towards the perfect patient and a little bit more towards the ones with the kind of barriers we have.” (P7)
		Make session more interactive, for example increased opportunity for role play amongst nurses so they could practice the script and potential scenarios “I think [the] education can be improved with including more interactive sessions, like let nurses role‐play on themselves when you cover the programmes.” (P2)
		Demonstration of intervention with actual patients, for example researcher delivers education and staff provided with the opportunity to observe “I’d probably just sort of say, ‘Hey, I’m going to go in and have a chat with Mrs Jones about this. Come along and just have a listen.’…it’s the sort of thing that’s best – I think it’s best seeing in person” (P7)
3 Teaching methods	Demonstration of ways to approach two different patients	More interactive sessions, for example small group discussion following viewing of the videos. “I think education can be improved with including more interactive sessions, like let nurses role‐play on themselves when you cover the programmes. Like, instead of working, a lot of videos.” (P2)
		Provision of ongoing support and feedback from research leads “…maybe two in‐services are the best way to do it. As in, ‘OK, this is the evidence behind it, this is what we want you to do’, and then the in‐service being, ‘This is an example of how to do it’, and so they’re giving us an opportunity to have a go at doing it, maybe role‐playing or something, and giving feedback …so we’re a bit more confident that we’re …delivering the education the right way.” (P3)
		Consistent discussion and reinforcement required during follow‐up sessions “…for the videos that we watch online and then have a 15‐minute discussion, would’ve been better.” (P5)
4 Educational resources	Accessibility of cognitive aids such as scripts. “…we had the laminated sheets pretty much everywhere, I think. For a while we had them stuck to our handover boards. Yeah, so they were very readily available, and it did give you the confidence of, you know, oh, that’s right, we’ll go in and just have that chat with them.” (P7)	Access to online learning outside of education sessions. I think if I’d known that I was able to go back and review it, that would have been helpful for me, just to cement my understanding of it. (P3)

### 4.1 Presage components: The context

#### 4.1.1 Relationship with stakeholders

##### Consultation and Pre‐course preparation

Some participants recommended that in the weeks preceding implementation of the intervention, there should be induction and orientation. One participant suggested this could be achieved by regular reminders during ward handover to foster engagement and facilitate assimilation with the new patient education approach.

#### 4.1.2 Management support

##### Time available for education

Participants agreed that a 1‐h session on its own was not long enough and an extended initial education session would have been more beneficial. Additionally, participants perceived that the initial session needed to be followed up with one or more refresher sessions to reinforce the process.

#### 4.1.3 Learning environment

##### Online versus face to face

Some participants valued the flexibility offered by the opportunity to complete the training online in their home environment. Others who attended online felt face‐to‐face delivery would have been more valuable and would provide the ability to do more interactive activities such as role‐playing. Another participant suggested the videos and PowerPoints should have been accessible online, followed by face‐to‐face discussions. Others suggested that face‐to‐face would provide the ability to do more interactive activities such as role‐playing.

#### 4.1.4 The learners

##### Interprofessional collaboration

There was no statistically significant difference between participants' views on interprofessional collaboration before and after the education intervention (Table 2). Whilst one participant commented that following the education session existing intraprofessional (within profession) and interprofessional (between professions) communication had been further reinforced, another claimed that there had been no change as they already worked collaboratively.

### 4.2 Process components: The education programme

Participants' views on the education programme are presented in Table 3. Mean scores ranged from 3.71 to 4.04 for questions related to the content of the education (Table 3).

#### 4.2.1 Content: Evidence‐based practice

Mean scores were mostly above 4.00 for participants' views on evidence‐based education for falls prevention (Table 4). The only statistically significant increase (*p <* .05) was participants feeling more prepared to educate patients on falls prevention posteducation. The only question that scored below a mean of 4.00 both pre‐ and post‐education was participants believing they could overcome the barriers to implementing patient education of falls prevention. One participant reported increased confidence and motivation to implement the intervention knowing that the care they were providing was evidence‐based. However, another commented that they were already confident that the care they were giving was evidence‐based and therefore the education did not influence their practice.

#### 4.2.2 Teaching approaches and activities

One participant found the simulation videos demonstrating the intervention to be beneficial. Conversely, another felt that the scenarios needed to be more realistic to the patients they would be delivering the education to, such as demonstrating the intervention applied to patients with, for example hearing difficulties, language barriers, or cognitive barriers.

#### 4.2.3 Teaching methods

It was recommended that demonstration of the intervention with actual patients on the ward would have been beneficial, providing participants with the opportunity to observe how the education should be delivered. Another suggestion was to make the education session more interactive, for example using a role‐play scenario, which would allow participants to practice delivering the script before educating their patients. Some participants felt that a one‐off education session was not enough and there should be ongoing support and feedback from clinical leads on the ward throughout implementation. Others suggested there should be follow‐up sessions to allow for further discussion and additional reinforcement of the implementation of the intervention.

#### 4.2.4 Educational resources

Many participants appreciated the availability and accessibility of cognitive aids on the ward such as the patient education scripts, which served as reminders to conduct the SRP intervention. Others proposed that the videos of the education session be obtainable for access outside of the education session, suggesting that they were not aware that these resources were available.

## 5 DISCUSSION

**FIGURE 2:**
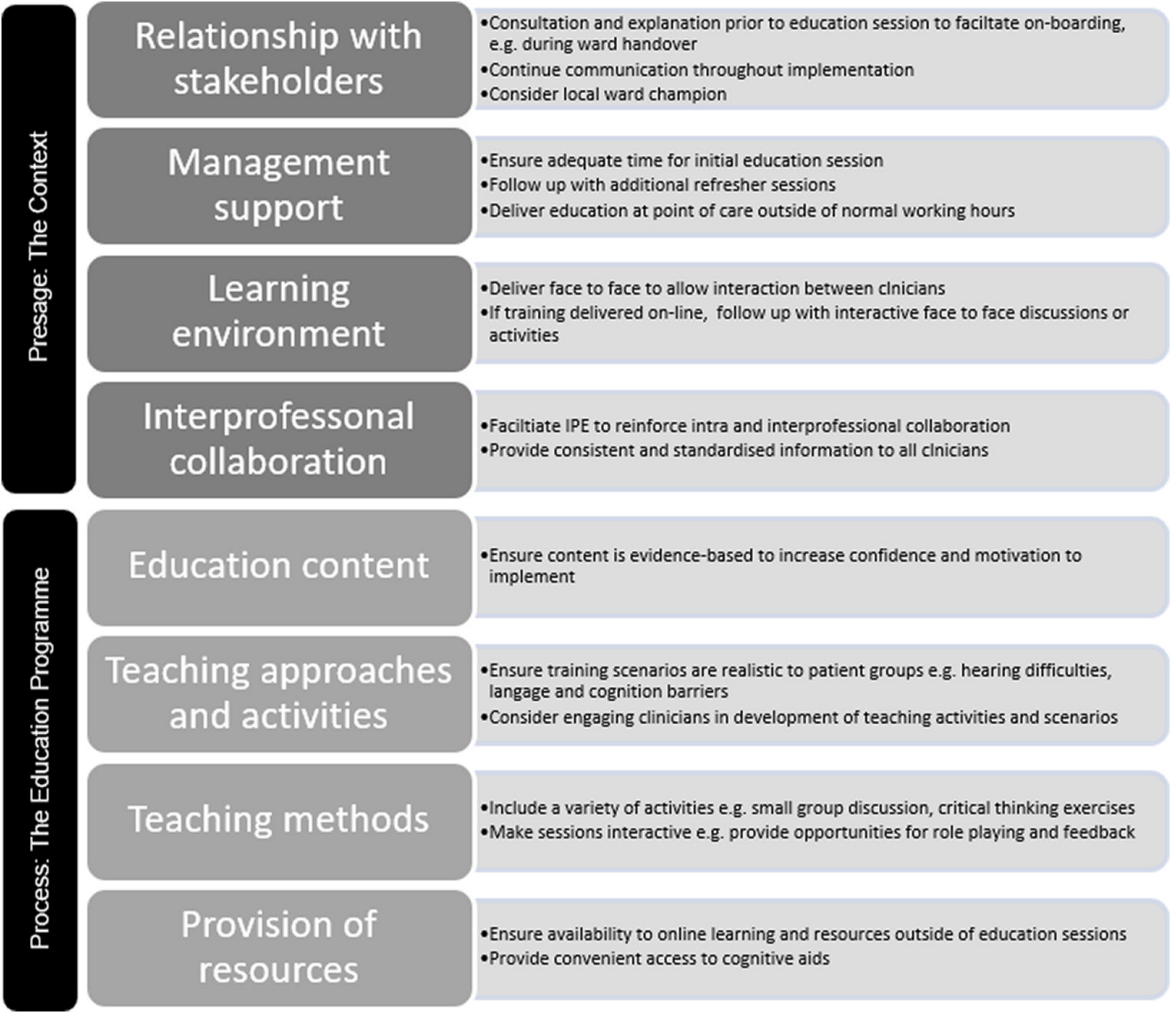
Recommended strategies to enhance implementation of a clinician IPE programme for delivery of falls prevention education

This hospital‐based study highlights important factors influencing the implementation of an interprofessional education programme to educate patients on falls prevention. Recommended strategies to overcome barriers are summarized in Figure 2. Application of the 4P model optimized the evaluation and reporting of the education intervention.

### 5.1 Presage components: The context

The findings suggest that a pre‐requisite for implementing an effective education intervention is building relationships with health professionals and clinical managers (Damschroder et al., 2009). It is essential to continuously build the case for change (McKenzie et al., 2019) and promote the benefits of patient education in falls prevention. Regular and effective communication should be provided throughout implementation to ensure sustained staff engagement and enthusiasm (Sarkies et al., 2017). Leadership engagement has been identified as being essential for implementation of research findings into practice (Damschroder et al., 2009). Other research in falls prevention education identifies the need for clinical leaders to regularly connect with their staff and support them during implementation (Shaw, Kiegaldie, & Morris, 2021). One way of achieving this could be to consider appointing local ward‐based champions to disseminate the key messages, assist frontline health professionals with implementation, and act as a point of contact between the health professionals who are delivering the education and the project leaders (Ayton et al., 2017).

Patient education has been found to be the single most important evidence‐based strategy for falls prevention (Heng, Jazayeri, et al., 2020; Webster et al., 2021), which demonstrates that healthcare organizations need to place greater value in educating healthcare professionals to undertake this task. In our study, some participants asserted that the initial education was insufficient in length. Previous research on the implementation of evidence‐based guidelines for falls prevention demonstrates that longer training fosters clinicians' confidence increases satisfaction and prepares them to more effectively educate others (Shaw, Kiegaldie, Morris, & Jones, 2021). Additionally, our findings indicated that a one‐off education session may not equip staff with the necessary skills to translate knowledge into practice, which accords with other research in falls prevention education (Shaw, Kiegaldie, & Morris, 2021, Shaw, Kiegaldie, Morris, & Jones, 2021). Previous research has found that whilst allied health professionals know the importance of evidence for changing their practice, they often feel ill‐equipped or under‐supported to implement this (Wenzel et al., 2020). Participants expressed a desire for additional refresher sessions, including consistent discussion on the topic and continual reinforcement. Regular interaction and small group interactive training sessions have been found to be necessary factors in clinical guideline implementation (Fischer et al., 2016).

There were several challenges delivering the IPE both online and concurrent with face‐to‐face delivery, which may partly explain why those attending via online education felt less engaged. These findings demonstrate the need to deliver the education separately to ensure that each participant is fully engaged and personally involved. This allows for greater responsiveness to participants' learning needs (Shaw, Kiegaldie, & Morris, 2021; Wensing et al., 2020).

Delivering a single education workshop did not result in a statistically significant change in clinicians' views on interprofessional collaboration. This may be due to participants already rating their interprofessional collaboration highly prior to the education. A four‐hour interprofessional falls prevention workshop that included interactive strategies and 2 h of individualized team planning, resulted in statistically significant increases in knowledge and confidence in skill performance (McKenzie et al., 2017). This reinforces the value of interprofessional collaboration and suggests a more interactive approach and extended interprofessional education may have further enhanced participants' perceptions of the importance of interprofessional collaboration for falls prevention.

### 5.2 Process: The education programme

Our education intervention did not significantly change participants' views on evidence‐based practice. This may be because they felt the organization already fostered a culture where the use of evidence was valued, and participants perceived that the care they were delivering was already evidence‐based. Evidence‐based education programmes for health professionals are critical for providing quality patient care (Lehane et al., 2019; Shaw, Kiegaldie, & Morris, 2021; Shaw, Kiegaldie, Morris, & Jones, 2021). Implementation of evidence‐based training in falls prevention has been shown to increase learning and produce practice changes (Shaw, Kiegaldie, Morris, & Jones, 2021).

The use of simulated participants to demonstrate the delivery of the patient education was valued by some participants who felt it improved their knowledge and skills. However, others suggested that to change behaviour, teaching strategies needed to be more interactive with role‐playing and feedback. Whilst this type of delivery is more difficult during the COVID pandemic, it still appears to be the more desired approach amongst clinicians. Tailoring education interventions at the unit level, engaging clinicians in the development process and involving them throughout the process, could assist with achieving behaviour change and a positive attitude towards implementation (Oakman et al., 2021). Active learning methods are more likely to facilitate the development of logical reasoning (Von Colln‐Appling & Giuliano, 2017), reflective thinking (Colley et al., 2012) and the effective translation of evidence‐based practice (Horntvedt et al., 2018). With more time available for training, teaching strategies such as problem‐based learning, group discussions and critical thinking through case‐based learning could be employed.

Participants appreciated the availability of resources related to the implementation of the patient education such as laminated signposts and the education script. However, some participants were unaware that recordings of the education session were available for further review. The availability of educational materials such as the audio‐visual recording needs to be made more explicit and reminders provided in ward handover. Healthcare professionals also need to have easy access to digital technologies such as tablets onwards to be able to revise the education intervention whenever they required.

## 6 CONCLUSION

Educating healthcare professionals to prevent hospital falls is an important activity warranting dedicated time and resources. This study showed that a single education session is not enough for lasting changes in health professional knowledge and patient falls prevention behaviours. The implementation sciences literature, coupled with these findings, reinforce the need for co‐production of falls education, involving the patient and interprofessional team. Developing and evaluating health professional education programmes using the 4P model of education design, ensures all elements of the teaching context, student approaches to learning and the outcomes of learning are considered.

## AUTHOR CONTRIBUTIONS

**Louise Shaw:** Conceptualization, methodology, validation, formal analysis, investigation, resources, data curation, writing—original draft, and writing—review and editing. **Debra Kiegaldie:** Conceptualization, methodology, validation, formal analysis, investigation, resources, and writing—review and editing. **Hazel Heng:** Conceptualization, writing—reviewing and editing. **Meg E. Morris:** Funding acquisition, project administration, and writing—review and editing, supervision.

## CONFLICT OF INTEREST

The authors declare that they have no competing interests. The authors alone are responsible for the content and writing of this paper.

## ETHICS STATEMENT

Research Ethics Committee approval was obtained from La Trobe University’s Health and Engineering College Human Ethics Sub‐Committee (HEC21023).

## Supporting information


Supplementary Material

## Data Availability

Data from the study are available from the corresponding author on reasonable request.
