# Supplementary material for: Interprofessional education to implement patient falls education in hospitals: Lessons learned
Source: Nurs Open. 2022 Jun 23;10(1):36–47. doi: 10.1002/nop2.1276 (PMC9748046; doi:10.1002/nop2.1276)
Supplement: Supplementary file 1 — Supplementary Material [file NOP2-10-36-s001.docx]

**SUPPLEMENTARY MATERIAL**

**Appendix S1.**

# A conversation with patients for the prevention of falls in hospitals

Introduction: Prior to this conversation, you should have already introduced yourself to the patient, built rapport and asked the patient to read the Healthscope falls brochure.

You should be aware of the patient’s history, any issues that they’re presenting with, and understand how it might impact on their risk of falls while in hospital.

This script is meant to be a guideline, and you may find that you will need to be flexible in order to respond to the patient’s needs.

| **Step 1: Identifying the ‘leverage point’ (patient ‘buy in’) and linking back to falls prevention** |
| --- |
| - What would you like to get out of this hospital stay? (Potential answers: To get better, to go home, to improve, feel better etc) - That’s a great goal. We want to help you [get better, go home etc] as well. One way of doing that is to stop any falls from happening. Do you think there is a chance you might fall in hospital? - Depending on the patient’s reaction, respond appropriately. Here are some example responses:   - Draw attention to the patient’s individual risk factors (e.g. post-surgery effects, dizziness, toileting urges, reduced strength, unfamiliar place)   - Reassure patient if they feel anxious or fearful of falling   - Draw out what the patient thinks is their main falls risk factor - Our research has shown that when people come into hospital, they have a risk of falling, because they’re not well. - If you fall, you could hurt yourself, which might mean staying longer in hospital. So, to get home and live independently, it is important to avoid falls. - The brochure we’ve given you has lots of useful information about preventing falls. Have you had a read yet? Do you have any questions? (Clarify any information here, and point out specific strategies for individual patients e.g. hypotension, reduced strength, diuretics) |
| **Step 2: Setting a goal** |
| - Something we have found that helps as well, is being prepared. Let’s come up with some ways to keep you safe and to [get better/go home/link with main goal]. - What are some things you can do whether by yourself or with our help to prevent you from falling while you’re here in hospital? |
| **Step 3: Follow up (This may take place at any point during the patient’s stay. Either you or another clinician may check in with the patient. You may follow up with another patient whom you did not see for step 1 and 2)** |
| - Have you been able to do the things you mentioned before to prevent falls? |

**Appendix S2.**

# EMPOWER Trial Process

| **Step 1:** Deliver education conversation 24 hours after patient is admitted to the ward |
| --- |
| **Step 2:** Immediately place laminated sign into the front outside plastic pocket of the patient’s bedside folder |
| **Step 3:** Throughout the patient’s stay, if you see the laminated sign, please have follow-up conversations with them |
| **Step 4:** Remove the laminated sign and place back into tray once patient is discharged |
